# The role of asymmetry and volume of thrombotic masses in the formation of local deformation of the aneurysmal-altered vascular wall: An in vivo study and mathematical modeling

**DOI:** 10.1371/journal.pone.0301047

**Published:** 2024-06-13

**Authors:** Denis Tikhvinsky, Maria Maus, Anna Lipovka, Nikita Nikitin, Rostislav Epifanov, Irina Volkova, Rustam Mullyadzhanov, Alexander Chupakhin, Daniil Parshin, Andrey Karpenko

**Affiliations:** 1 Department of Mathematics and Mechanics, Novosibirsk State University, Novosibirsk, Russia; 2 Department of Vascular Pathology and Hybrid Surgery, Meshalkin National Medical Research Center, Novosibirsk, Russia; 3 Laboratory of supercomputing and artificial intelligence in energetic technologies, Kutateladze Institute of Thermophysics SB RAS, Novosibirsk, Russia; The British University in Egypt, EGYPT

## Abstract

Currently, the primary factor indicating the necessity of an operation for an abdominal aortic aneurysm (AAA) is the diameter at its widest part. However, in practice, a large number of aneurysm ruptures occur before reaching a critical size. This means that the mechanics of aneurysm growth and remodeling have not been fully elucidated. This study presents a novel method for assessing the elastic properties of an aneurysm using an ultrasound technique based on tracking the oscillations of the vascular wall as well as the inner border of the thrombus. Twenty nine patients with AAA and eighteen healthy volunteers were considered. The study presents the stratification of a group of patients according to the elastic properties of the aneurysm, depending on the relative volume of intraluminal thrombus masses. Additionally, the neural network analysis of CT angiography images of these patients shows direct (*r* = 0.664271) correlation with thrombus volume according to ultrasound data, the reliability of the Spearman correlation is *p* = 0.000215. The use of finite element numerical analysis made it possible to reveal the mechanism of the negative impact on the AAA integrity of an asymmetrically located intraluminal thrombus. The aneurysm itself is considered as a complex structure consisting of a wall, intraluminal thrombus masses, and areas of calcification. When the thrombus occupies > 70% of the lumen of the aneurysm, the deformations of the outer and inner surfaces of the thrombus have different rates, leading to tensile stresses in the thrombus. This poses a risk of its detachment and subsequent thromboembolism or the rupture of the aneurysm wall. This study is the first to provide a mechanistic explanation for the effects of an asymmetrical intraluminal thrombus in an abdominal aortic aneurysm. The obtained results will help develop more accurate risk criteria for AAA rupture using non-invasive conventional diagnostic methods.

## Introduction

The aneurysm wall is a heterogeneous anisotropic structure, which, apparently, is associated with a different degree of degradation of the extracellular matrix within one vascular segment. Currently, these processes are not fully understood. It appears that both general metabolic processes of the body and local hydrodynamic changes play a role in abdominal aortic aneurysm AAA formation [1]. Aortic aneurysm rupture typically occurs suddenly in most cases, often leading to a fatal outcome without urgent surgical intervention. Rupture of an aortic aneurysm occurs in most cases suddenly and ends with a fatal outcome without urgent surgical intervention. Thus, the mortality from AAA rupture reaches 80%, constituting 2% of the total number of deaths in the population [2, 3]. Hospital mortality in this condition reaches 65%, and the postoperative mortality rate—41,65% [4]. In this context, planned surgical intervention is considered the most viable option for treating aneurysms, necessitating increased attention to the pathology itself. The indication for elective surgery is the maximum transverse diameter of the aorta, which, at the moment, is defined as the main risk factor for rupture of the pathologically altered aorta. [5]. At the same time, it is known that aneurysm ruptures occur in a number of patients before the threshold values recommended for surgical correction are reached, just as not all aneurysms that have reached the recommended operating diameter in size are ruptured [6, 7]. Given that this pathology is most often prevalent in patients of older age groups, elective and emergency surgeries are accompanied by an increased risk of complications, often being the main cause of postoperative deaths for many patients. In this regard, there is an urgent clinical need to identify predictors of growth and the risk of aortic aneurysm rupture for optimal planning and preparation of surgical correction for patients [8, 9]. Attempts to isolate local risk factors for aortic rupture did not lead to the expected results. With the advent of the possibility of calculating the peak shear stress on the aortic wall and comparing it with the risk of rupture, in some works, confirmation of this hypothesis was obtained—the minimum shear stress in the area of the alleged aneurysm rupture [10–12], and in some it was refuted [13]. Consequently, it has been suggested that peak shear stress of the wall and its strength are independent variables. Due to the fact that method of *in vivo* calculation of the strength of the vascular wall has not been developed, there is a need to search for new indirect markers of its assessment. It is possible that it may depend on the peak shear stress of the vascular wall and the amplitude of its elastic deformation. In some studies [14], using numerical modeling of an idealized AAA, an attempt is made to assess the influence of an intraluminal thrombus on the risk of aneurysm rupture, but the methods used did not allow for an assessment of the influence of the geometric characteristics of the thrombus.

Aortic aneurysm rupture is rooted in a decline in the strength properties of a pathologically altered vascular wall due to the degeneration of the elastic framework and an increase in systemic arterial pressure [15]. In recent years, the evaluation of the mechanical properties of the pathologically altered aortic wall has been increasingly carried out using ultrasound. Reflected ultrasonic waves enable the observation of local movement of the vascular wall in real time, dependent on the pulse wave cycle. An in-depth analysis of these deformational changes may reveal additional factors that play a role in the rupture of the aortic wall. Undoubtedly, one of these factors is the intraluminal thrombus, the influence of which has been repeatedly confirmed [16, 17]. While previous observations have noted the influence of the symmetry of the intraluminal thrombus location [18]and its density [19, 20], a mechanical representation of this influence on both aneurysm rupture and the occurrence of thromboembolic complications has not been presented. Moreover, given the operator-dependent results of ultrasound measurements [21, 22], modern medicine needs either alternative methods for assessing deformation or the development of operator-independent protocols for ultrasound measurements of aortic aneurysm deformation. Given the variability in individual numerical calculators for the risk of abdominal aortic aneurysm rupture, the most reliable approach to solving the problem facing the global community seems to be the use of a combination of mathematical modeling, development of approaches in ultrasound diagnostics and processing of medical image data using advanced machine learning algorithms to achieve maximum operator-independence of measurements.

The purpose of this study is to assess the influence of the volume and nd spatial distribution of thrombus masses within the AAA lumen on the nature of elastic deformations of the wall and lumen of the vessel. Mathematical modeling is employed to reveal the mechanism of their asymmetric location’s negative impact on outcomes: aneurysm rupture or thromboembolic complications. To perform the study, a combination of ultrasound techniques, numerical finite element modeling on idealized AAA configurations with intraluminal thrombus and spine, as well as machine learning methods for processing medical images were used.

## Materials and methods

### The main and control group

From 1st December 2022 till 10th February of 2023, in the research department of vascular and hybrid surgery of the Meshalkin National Medical Research Center 29 adult patients admitted with aneurysms of the abdominal aorta for planned surgical correction were examined by ultrasound. Preoperative ultrasound examination of the abdominal cavity and aorta was performed to clarify the nature of the aneurysmal expansion of the aorta, the presence of mural thrombus and the condition of the iliac arteries.

As a control group, 18 volunteers under the age of 30 with no aortic pathologies were examined Table 1. In this group, an ultrasound pulse histogram was evaluated in the middle part of the infrarenal aorta. The selection of patients and the control group was carried out with their voluntary informed written consent for inclusion in this study, and the study was carried out in accordance with the conclusion of the local ethical protocol №07-3 from 20 May 2022 Meshalkin National Medical Research Center. The study is registered on the portal clinical.trials.gov: NCT05634018.

**Table 1:** Clinical characteristics of volunteers and patients with AAA. Clinical characteristics of volunteers and patients with AAA.

Clinical signs	Healthy patients (control group)	Patients with AAA
1. Gender: men/women	50%/50%	94%/6%
2. Average age	30 years	67 years
3. Smoking	20%	47%
4. Arterial hypertension	0	86%
5. Myocardial infarction	0	33%
6. Stroke	0	0%
7. Obliterating atherosclerosis	0	13%
8. Atrial fibrillation	0	13%
9. AAA diameter up to 5 cm	0	22%
10. AAA diameter 5-7 cm	0	46%
11. AAA diameter over 7 cm	0	32%

### Ultrasound imaging methods

In order to assess the elastic properties of the altered vascular wall, a transverse scanning of the aorta was performed at the site of its greatest expansion in the B-mode, in the color Doppler mapping mode, and in the pulsed wave Doppler mode. The recorded images and the movement of the vascular wall were processed at the GE US-station with the EchoPAC software. Software installation and configuration was performed by an authorized GE dealer.

In this study a technique that was originally created to assess the deformation properties of the myocardium—Speckle-tracking, was used to measure the deformation of aorta. The point of this method is that the ultrasound module software uses video loops of myocardial sections, divides the myocardium into points with stable visualization—speckles, and then tracks each point over several cardiac cycles. We applied this technique in measurements carried out on the abdominal aorta. Using Vivid iq ultrasound unit of GE Healthcare using convex sensors c1-5-RS and sector sensors M5Sc-RS, a cross-sectional recording was made at the site of maximum aortic aneurysmal expansion. To improve the quality of tracking, the recording was carried out with a patient holding breath at the maximum exhalation for 2-4 cardiocycles. Speckle-tracking evaluation in the circular direction was the subject to analyse. To standardize the obtained results, we examined 18 volunteers under the age of 30 of both sexes. Scanning in this case was performed in the middle part of the infrarenal aorta.

**Fig 1:**
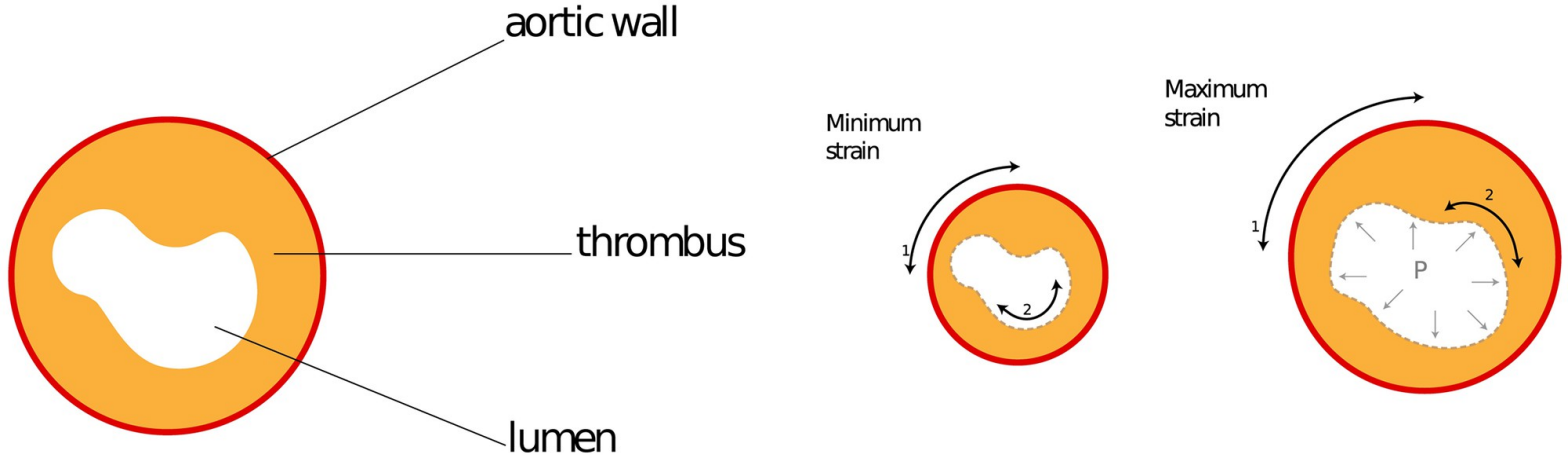
Strains of the wall and lumen. Illustration of the cross-section of the aneurysm (left); Illustration of the wall and lumen deformation (right).

**Fig 2:**
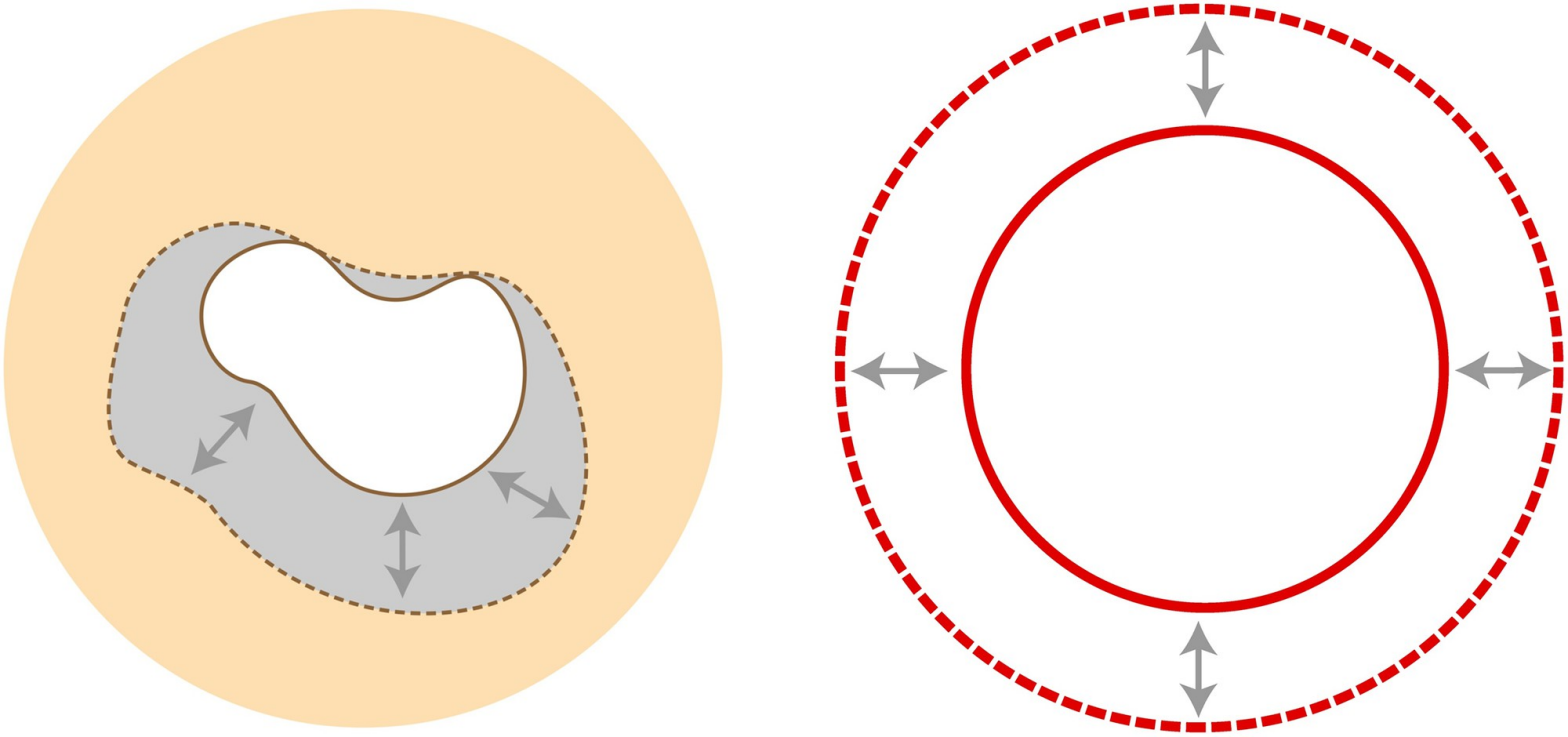
Strains of the wall and lumen. Change of the lumen, referred as the inner strain (left); change of the wall, referred as the outer strain (right).

In patients with aortic aneurysm, two zones for calculating elastic deformation were determined within one transverse scan (Fig 1)—the zone on the border of the vascular lumen free from thrombus and the zone corresponding to the vascular wall (Fig 2).

**Fig 3:**
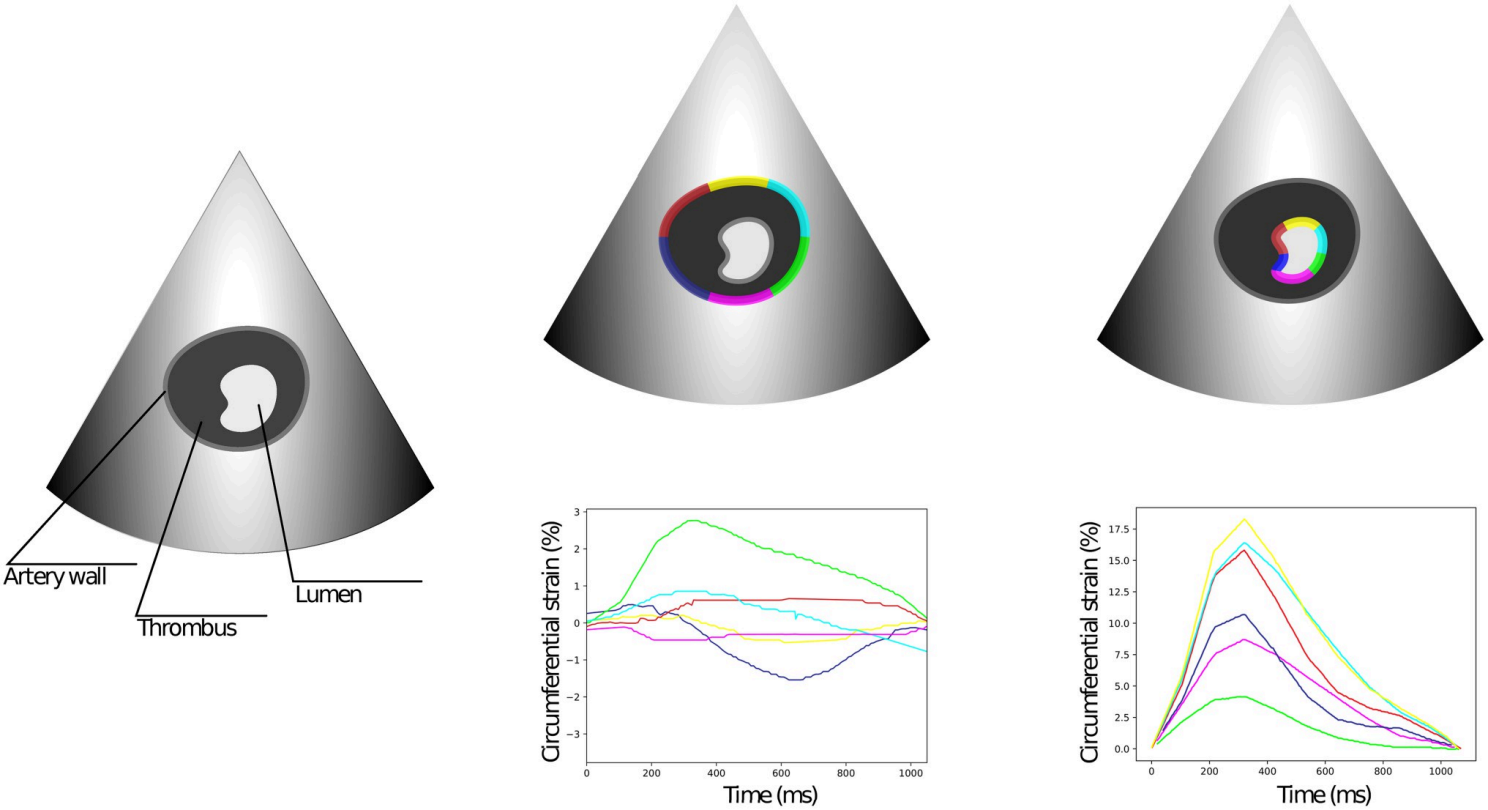
The concept of measurement in EchoPAC software. The depiction of the deformation measurement process, as performed by the EchoPAC software. The cavity of the aorta in transverse plane is visualised by the ultrasound. The aorta wall and lumen, formed by the thrombi, are captured and divided into 6 sectors. The deformations of each sector are given on the graphs. Change of the wall deformation is referred as the outer strain (left); change of the lumen is referred as the inner strain (right).

As a result of image processing, waves of elastic deformation of the vascular wall were obtained in 6 sectors during one cardiocycle (Fig 3). On the obtained histograms, the maximum global deformation of the vascular wall was estimated using the software package attached to the program.

### Computed tomography protocol and data analysis

Automatic calculation of the volumes of the aortic lumen and intraluminal thrombus masses in the analyzed patients was carried out by a neural network [23]. The used neural network has Unet architecture with resnext50_32x4d encoder. The neural network was trained on 30 CT images, for each of which triple-overlapping masks were prepared. The morphology of the aneurysm identified by the neural network is represented as a three-dimensional array equal in size to the original CT image. Each matrix voxel is assigned a value corresponding to the class of aortic lumen, thrombotic masses, or calcifications. To estimate the volume of the class of interest, the number of voxels of the class of interest was counted and the resulting number was multiplied by the volume of a single voxel.

### Methods of numerical calculation

**Fig 4:**
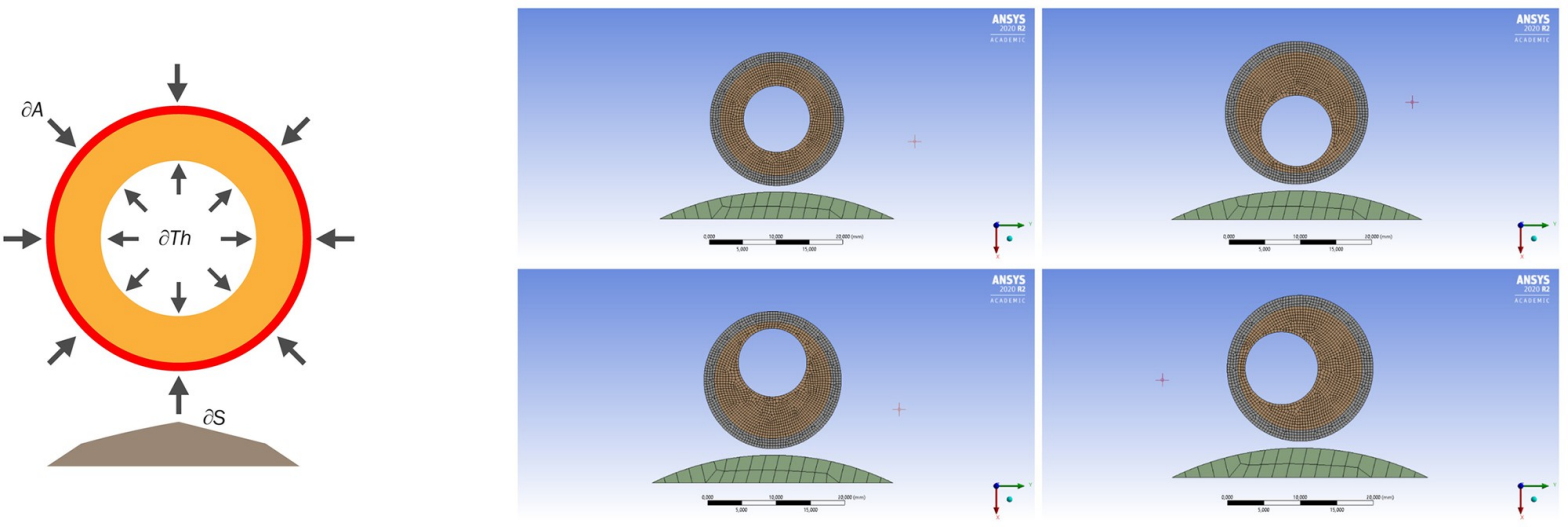
AAA slice model and boundary conditions. On the left—a schematic representation of the model with boundary conditions, On the right—options for the relative position of the spine, lumen and intraluminal thrombus.

Since it is currently possible to measure the deformation of the wall, thrombus, and lumen of the aorta in real time during diagnostic procedures, and also given the fact that all the studied aneurysms are different to some extent, the question arises of what they have in common. In the ANSYS Mechanical 2020 R2 package, loading was simulated from inside the aortic wall both with and without a thrombus attached to it. To simulate the interaction of the aortic wall with a thrombus and the spine, a 2D model was built. The wall was modeled as a ring with an outer diameter of 50 mm and a thickness of 2 mm. The thrombus in the lumen of the aorta was modeled by a second ring with an inner diameter of 17 mm and had contact with the inner surface of the vessel wall. The thickness of the inner ring, as well as the location of the lumen, varied depending on the amount of thrombus in the aorta and between four basic positions of the lumen: circular (the inner lumen corresponded to the center of the vessel), shifted to the spine, or anteriorly, as well as to the right or left of the central axis of the vessel with the formation eccentric in thickness of the simulated intraluminal thrombus (due to symmetry, only one numerical formulation is considered). The material of the aortic wall and thrombus was specified as isotropic and linearly elastic with the values of the shear modulus obtained experimentally. The value of thrombus Young’s modulus was taken from [17], and for the wall of the aneurysm, data was taken from [24]. Different cases of the location of the aortic lumen were considered: in the center, shifted 2.5 mm closer to the spine, shifted the same distance anteriorly from the spine, and shifted to the right or left side by the same distance from the axis of symmetry, respectively. Various lumen locations are shown in Fig 4 (right). To solve the problem in the simulated area, a structured prismatic grid was built. The characteristic cell size for the elastic walls of the aorta and thrombus was taken as 0.4 mm, and for the immobile and rigid spine, 2 mm. The problem was solved in a quasi-static formulation using the finite element method built into the package ANSYS [25]. Since we accepted the used materials of the vessel and thrombus as isotropic, and the problem is static, the static equations of elasticity are solved:

$$\begin{array}{c} -\nabla *((I+\nabla u)\Sigma (\text{E}(\text{u})))=0, \end{array}$$ (1)

where *u*—displacement,

$$\begin{array}{c} \text{E}=\frac{1}{2}\left(\nabla \text{u}+\nabla {\text{u}}^{T}+\nabla \text{u}\nabla {\text{u}}^{\text{T}}\right), \end{array}$$

$$\begin{array}{c} \Sigma (\text{E})=\lambda (\text{thE})\text{I}+2\mu \text{E}+\text{o}(\text{E}), \end{array}$$

**E**—Cauchy tensor, λ, *μ*—Lame coefficients, Σ—Piola–Kirchhoff stress tensor.

The equation of a plane contact problem of the theory of elasticity for two bodies:

$$\begin{array}{c} \int_{-a}^{a}ln\frac{1}{\left|\xi -x\right|}\sigma \left(\xi \right),d\xi =f\left(x\right),f\left(x\right)=\frac{f_{2}\left(x\right)-f_{1}\left(x\right)-\alpha +x\omega }{\theta_{1}+\theta_{2}}, \end{array}$$ (2)

where

$$\begin{array}{c} \theta_{i}=\frac{2(1-\nu_{i}^{2})}{E_{i}\pi },i=1,2; \end{array}$$

*σ*(*ξ*)—unknown contact stress, *α*—rigid displacement of one body relative to the second, *ω*—rotation of one body relative to another, *E*_*i*_- young modules, *ξ*—distance from the origin of coordinates to the place of action of the load, *a*—body size, *ν*—half-plane boundary displacement.

The boundary conditions in the contact problem were as follows: The loading area is the inner lining of the thrombus (*δTh*). A pressure of 100 Hg mm is applied to the entire surface of the loading area. On the outer surface of the aorta (*δA*) there is a constant pressure of 6 Hg mm, which corresponds to the average intra-abdominal pressure of an adult. The spine is fixed and immobile. Schematically, the boundary conditions are shown in Fig 4.

The results of numerical modeling were taken at 16 points: the aortic section was dissected by 4 lines, each of which was rotated by 45deg relative to the neighboring one, and measurements were taken on these lines on the surface of the aorta and the inner lining of the thrombus. To analyze and visualize the calculation results, standard values of the strength characteristics of the ANSYS Mechanical module were used: Total Deformation, Equivalent Elastic Strain [25].

### Statistics

For statistical data analysis, the open libraries sklearn, pandas of the Python language were used. To visualize the analyzed numerical indicators, the matplotlib and seaborn libraries were used.

## Results

### Results of analysis of CT and ultrasound data

A dataset was constructed from data from healthy volunteers and patients with AAA, including the following data: maximum and minimum deformation of the inner and outer rings, the percentage of the lumen occupied by a thrombus, and the location of the thrombus.

**Fig 5:**
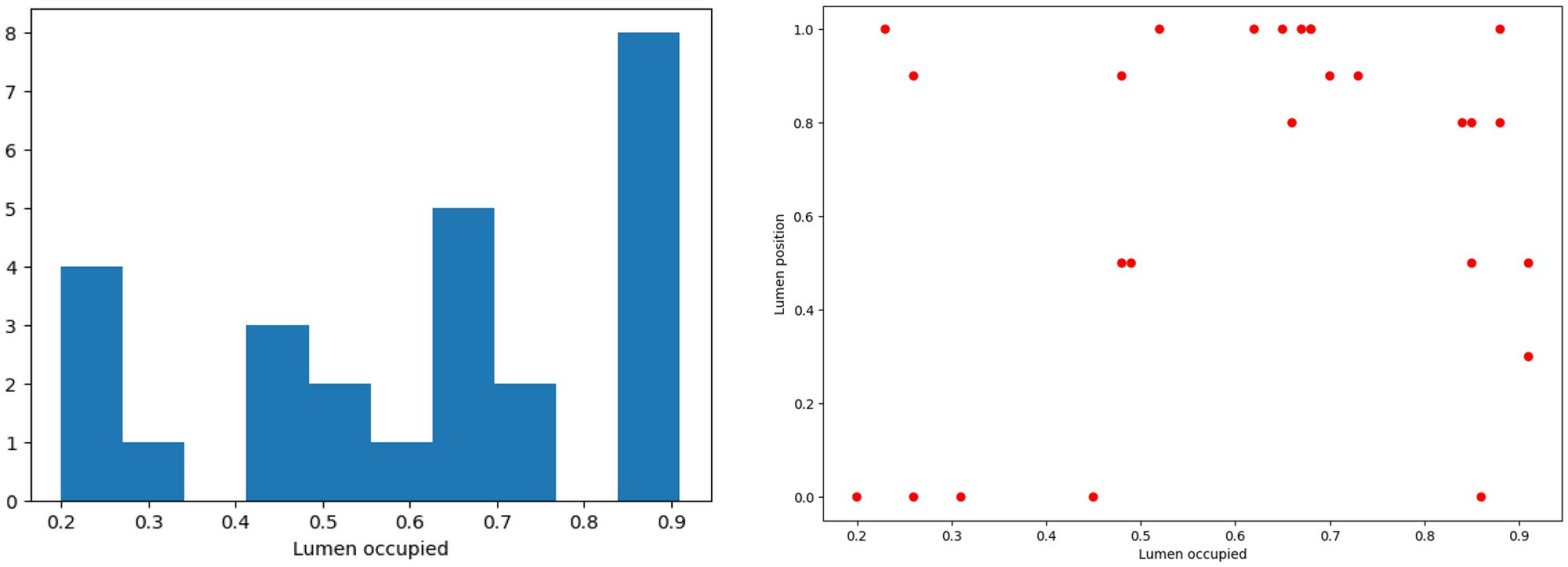
Data on thrombi. Left: distribution of percentages of thrombus mass in the lumen; right: The position of thrombus forming lumen depending on the thrombus mass. The position is the number in the range of 0-1, where 0—central position, 1—lumen is formed by the aortic wall from at least one side.

As we see from Fig 5 (left) the data is fairly well clustered by the percentage of thrombus in the lumen and has 4 clusters: 0.2 − 0.4, 0.4 − 0.6, 0.6 − 0.8, 0.8 − 0.99. In the future, we will use this fact of clustering when performing idealized numerical calculations so that the numerical formulation of the contact problem is as close as possible to the real clinical picture, and the demonstrated results are adequate.

**Fig 6:**
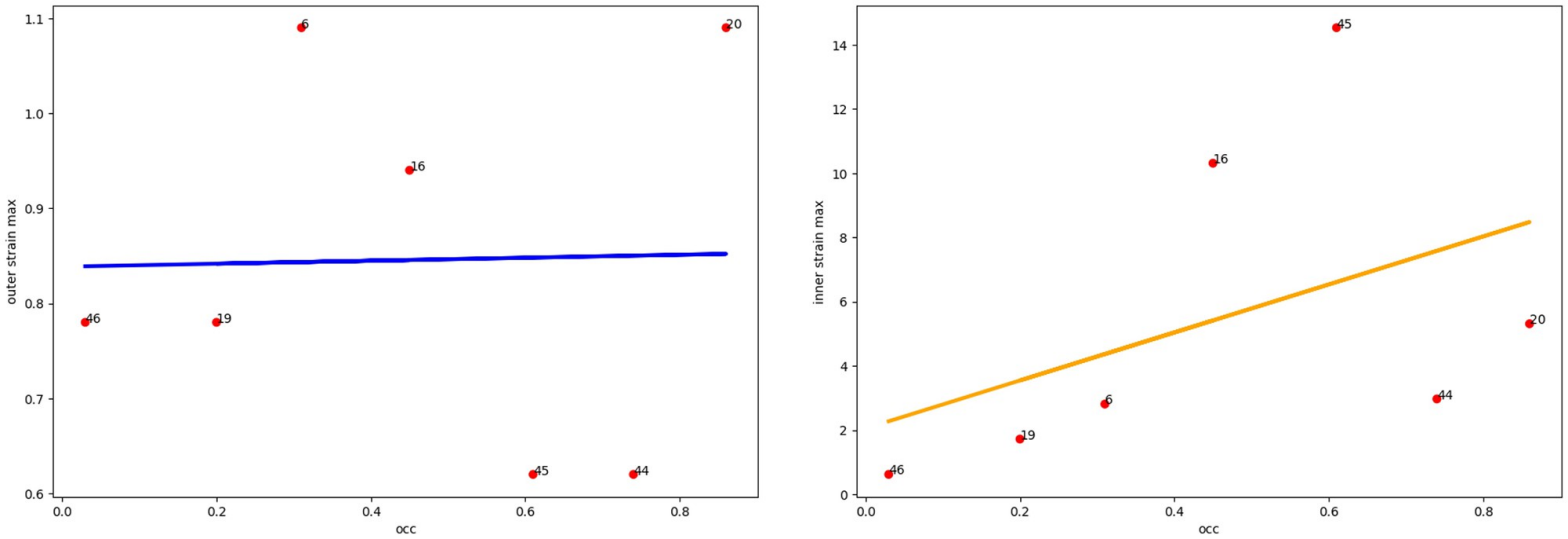
Thrombus boundary displacement trendline (central location). The maximum value of deformations of the vessel wall (left) and the inner lining of the thrombus (right) for the case of the central location of the intraluminal thrombus.

**Fig 7:**
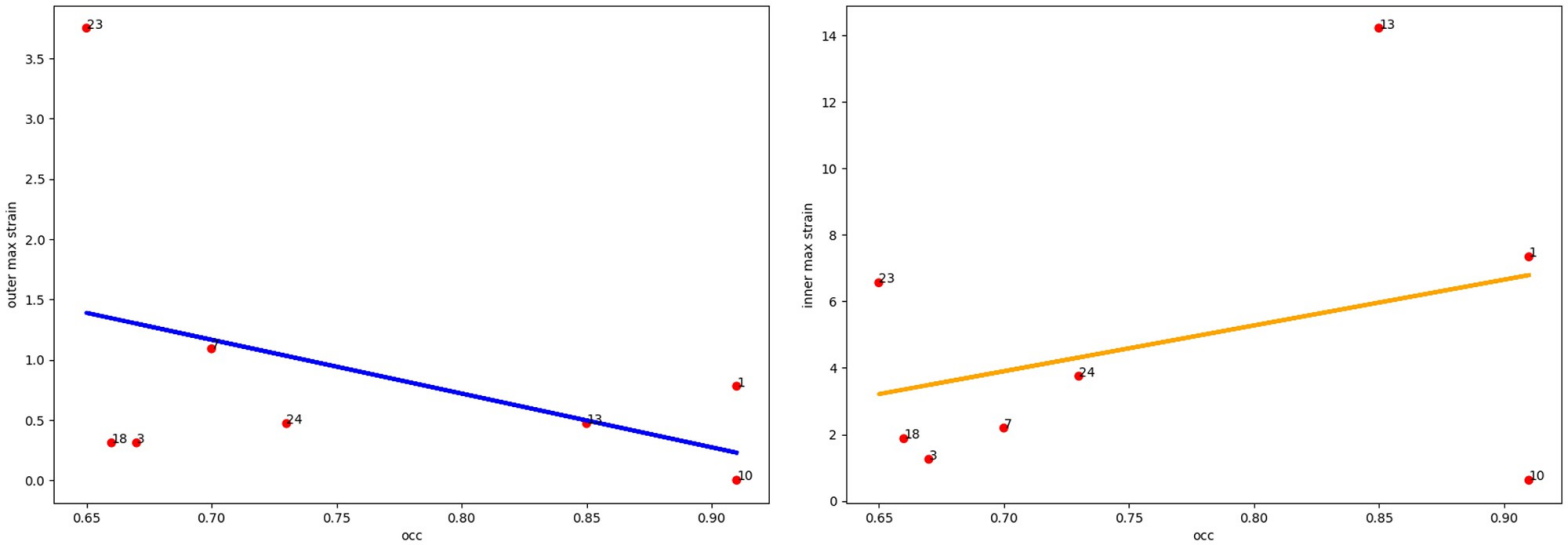
Thrombus boundary displacement trendline (peritoneuma location). The maximum value of deformations of the vessel wall (left) and the internal lining of the thrombus (right) for the case of an intraluminal thrombus displaced into the peritoneuma.

**Fig 8:**
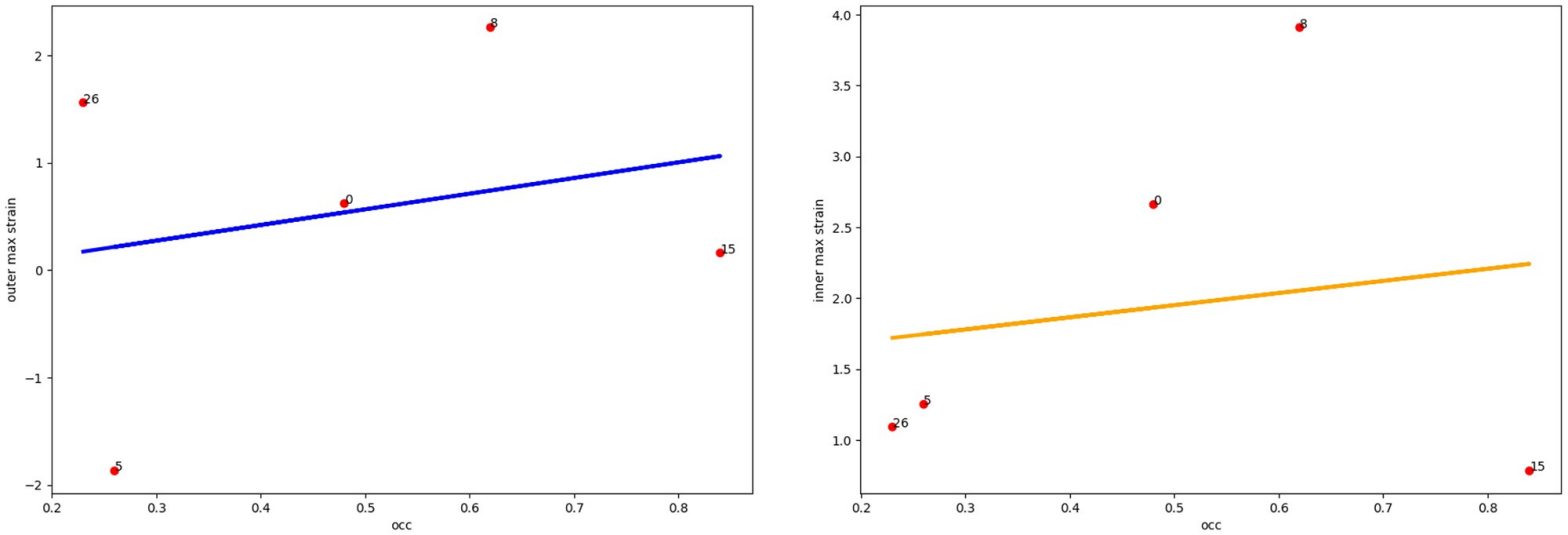
Thrombus boundary displacement trendline (spine location). The maximum value of deformations of the vessel wall (left) and the internal lining of the thrombus (right) for the case of an intraluminal thrombus displaced to the spine.

**Fig 9:**
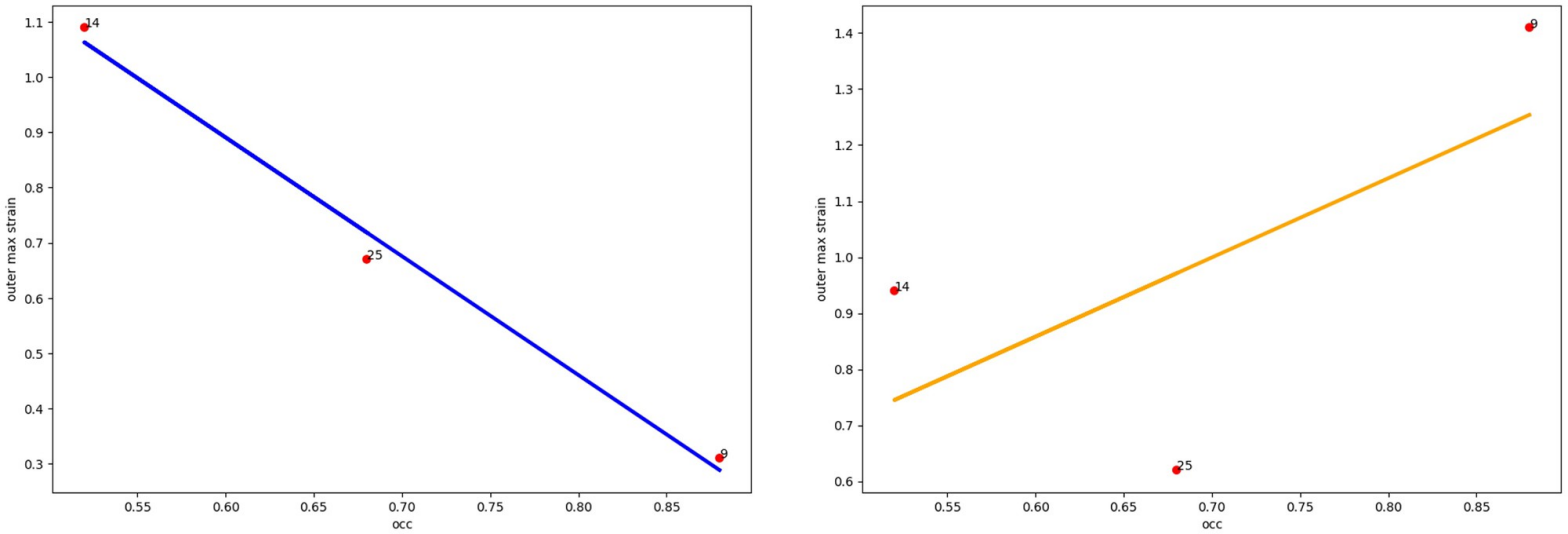
Thrombus boundary displacement trendline (left eccentricity location). The maximum value of deformations of the vessel wall (left) and the internal lining of the thrombus (right) for the case of an intraluminal thrombus displaced to the left.

**Fig 10:**
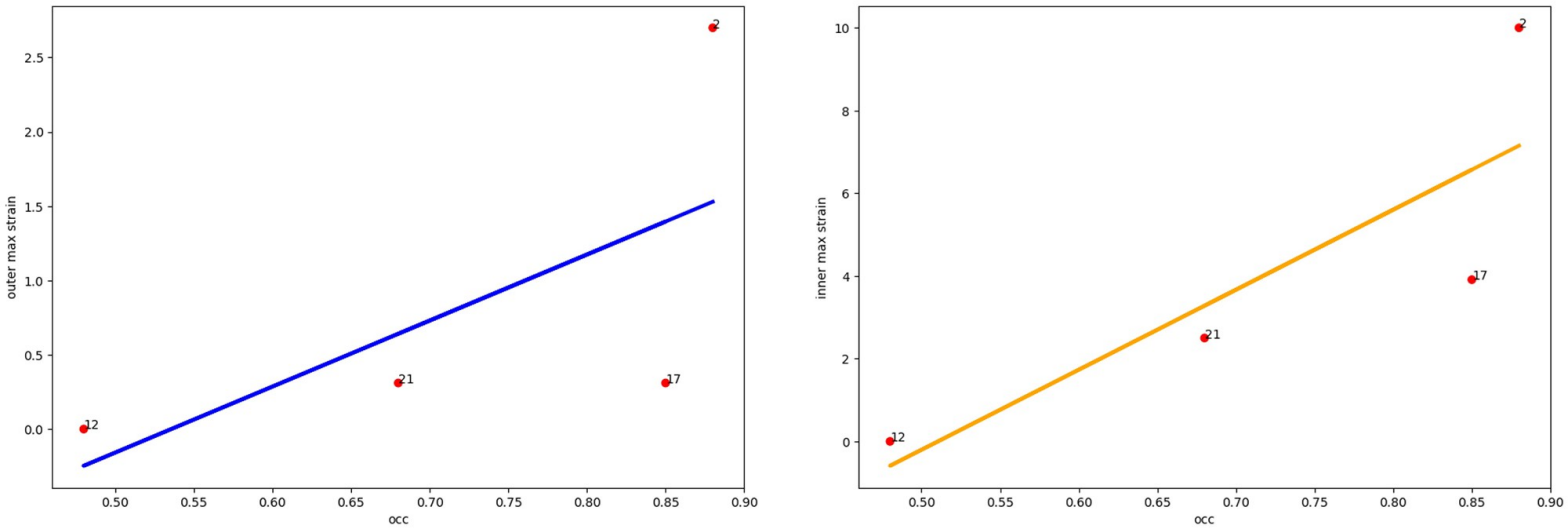
Thrombus boundary displacement trendline (right eccentricity location). The maximum value of deformations of the vessel wall (left) and the internal lining of the thrombus (right) for the case of an intraluminal thrombus displaced to the right.

Taking into account the fact that in the abdominal region the aortic aneurysm is surrounded by various structures with different elasticity (vena cava, spine), it is necessary to take into account the orientation of the thrombus in the lumen of the aneurysm (Fig 5 (right)). As we see from the Figs 6–10 for cases when the thrombus is displaced towards the vena cava or towards the spine, there is a divergent trend in the dependence of the amplitude of deformations of the inner and outer walls depending on the weaning of the thrombus. The outer wall becomes more and more rigid as the clot undergoes more and more deformations.

**Fig 11:**
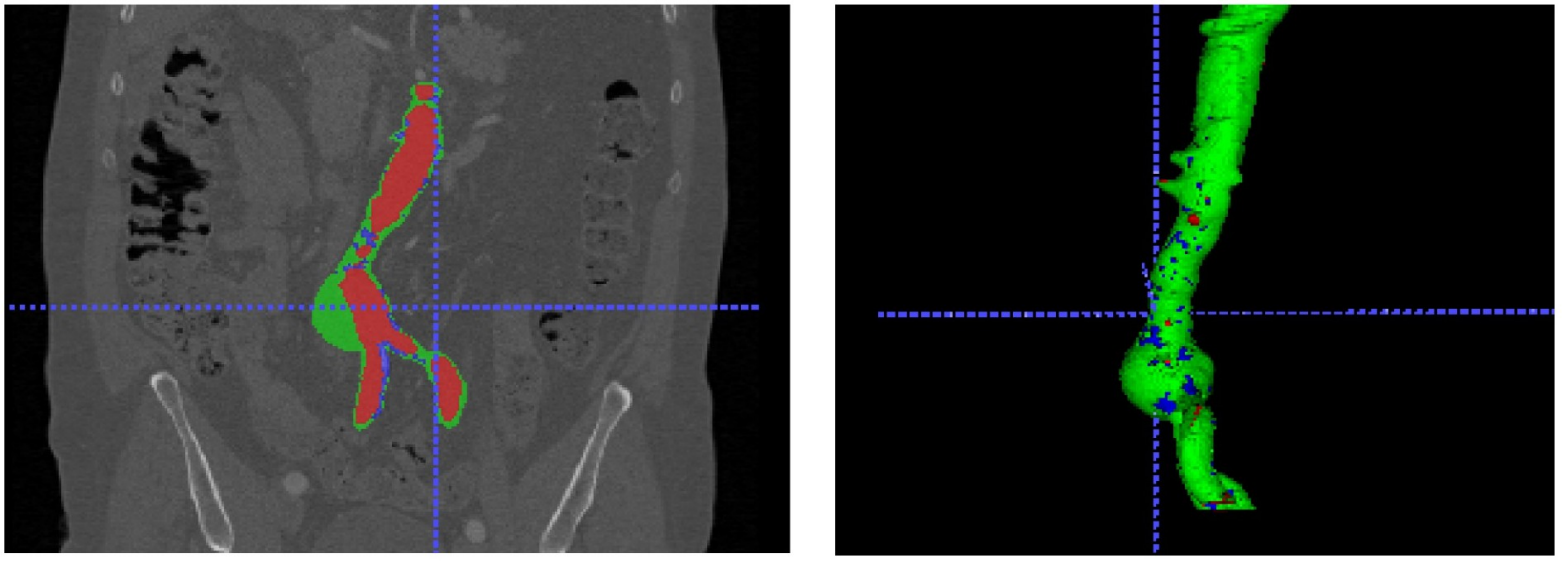
Segmentation result. Result of differentiated AAA segmentation of patient *03* with highlighting of the Vessel Lumen Area (red), intraluminal thrombus (green), and areas of calcification (blue). On the left in the sagittal projection, on the right—a three-dimensional image of the segmentation result.

Automatic segmentation was performed for a set of CT scans of 21 patients using our own program module (can be reached at https://aaa.nsu.ru/) [23] and ITK Snap (www.itksnap.org) [26], open-source, free software. Zones of intraluminal thrombus, vessel lumen and areas of calcification were identified, and the average density of thrombus masses in the AAA was also estimated. An example of the result of AAA segmentation of a real patient is shown in Fig 11.

**Fig 12:**
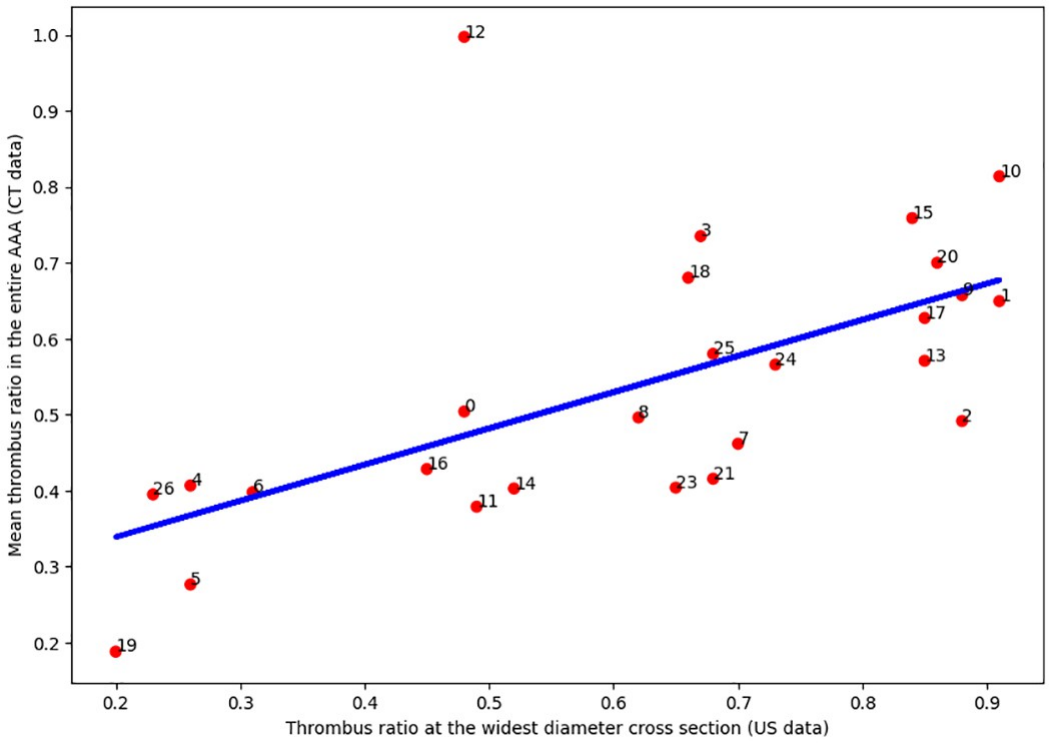
Correlation between US and CT data. Correlation between measured thrombus ratio through the US, and automatic segmentation from the CT scans.

The percentage of thrombus masses in the lumen of the AAA, as it turned out, is one of the most stable morphological factors throughout the AAA along the axis of the blood vessel. In particular, this percentage calculated from ultrasound data correlates well with the results of automatic segmentation. (Fig 12), where this indicator is already calculated as a volumetric characteristic according to the formula:

$$\begin{array}{c} Vol_{thr}=N_{thr}*Vol_{vox}, \end{array}$$ (3)

where *N*_*thr*_—the number of voxels based on the results of automatic segmentation, *Vol*_*vox*_—voxel volume from dicom image metadata.

**Fig 13:**
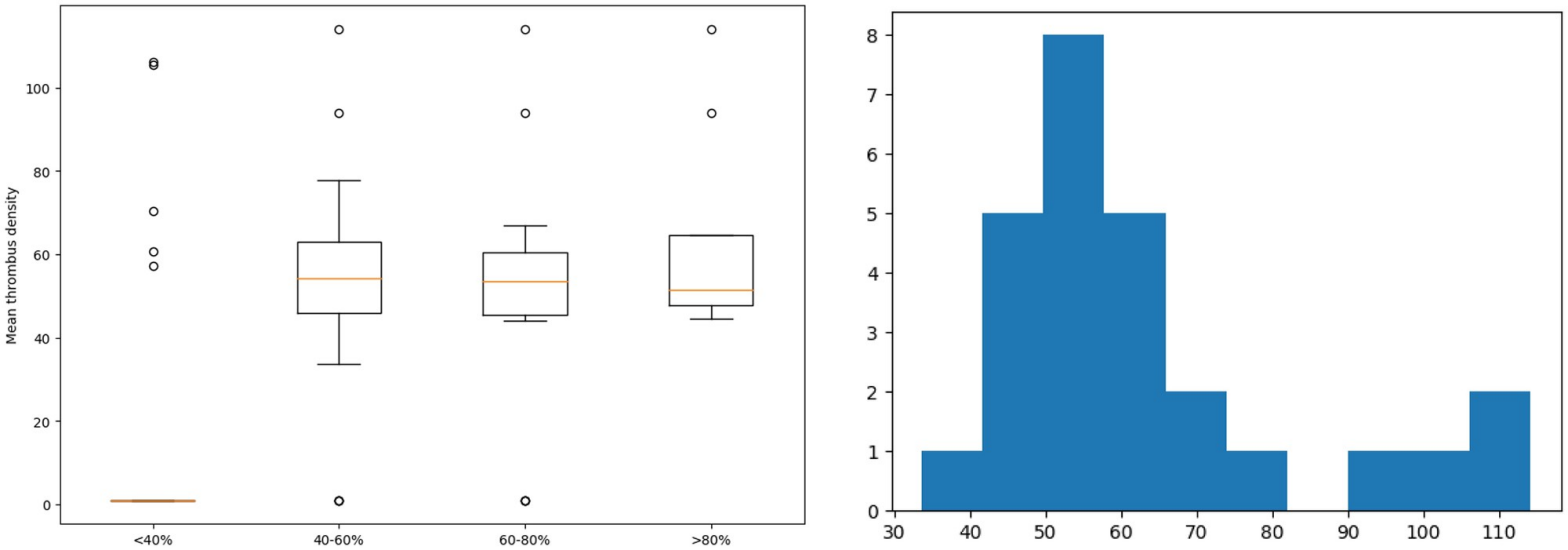
Distribution. Mean thrombus density for different clusters of thrombus masses (left); Distribution histogram of the mean thrombus density (right).

As we can see from Fig 13 (left) groups of patients with different percentages of lumen filling with a thrombus have similar median values of thrombus density, regardless of belonging to a cluster of a large or small amount of thrombus in the lumen.

Based on the fact that in terms of thrombus volume, our sample is not normally distributed (obviously from Fig 13 (right))—correlation analysis of the values of the percentage of thrombus masses by ultrasound and CT according to Spearman indicates a fairly strong correlation r = 0.664271 (reliability of the correlation p = 0.000215).

At the same time, we observe a distribution close to normal for a thrombus density not exceeding 80 units. As seen from Fig 13 (right) there are 2 clusters of patients for whom the thrombus density differs by an average of 2 times.

### Results of numerical simulation and qualitative data analysis

**Fig 14:**
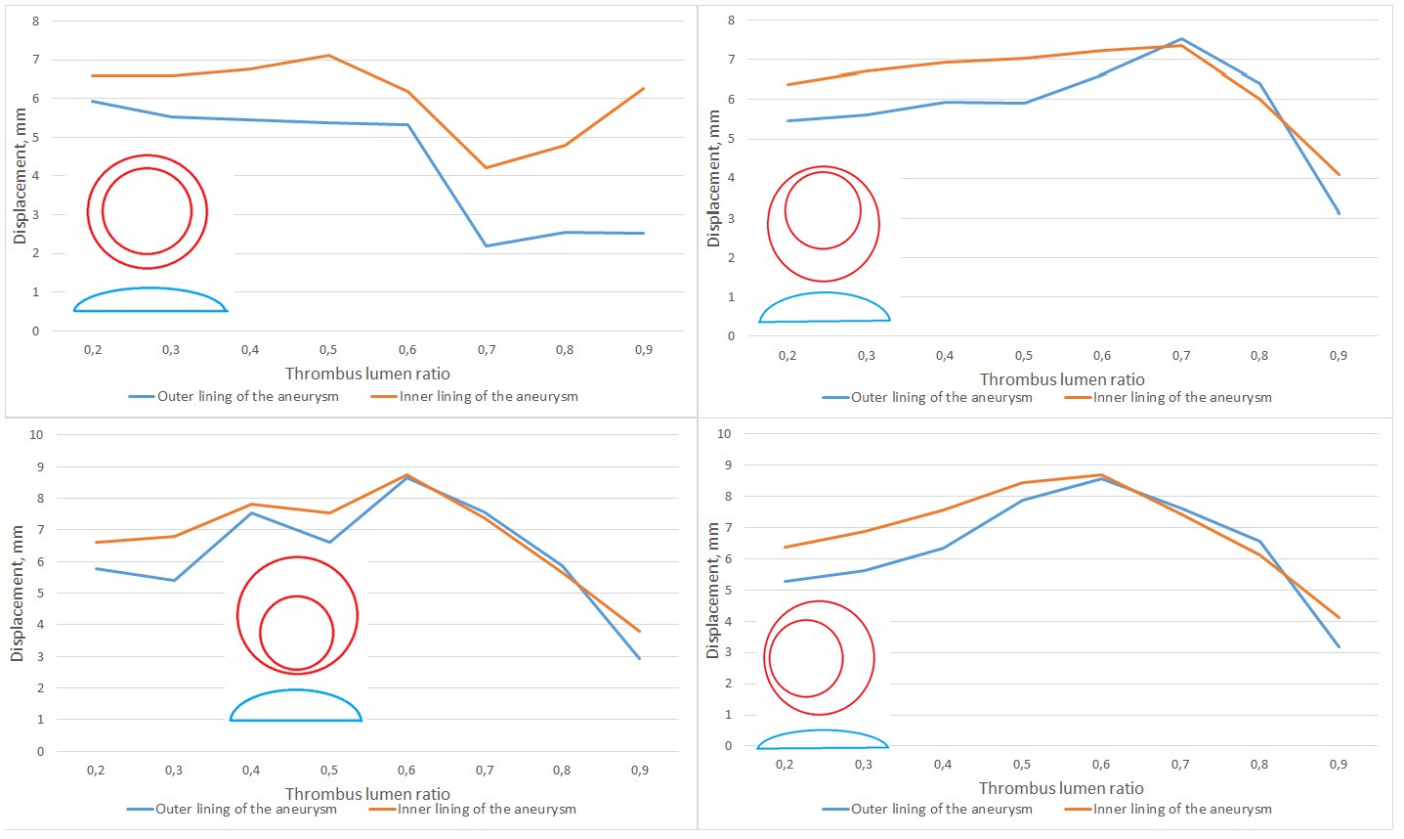
Displacement wall and thrombus. The maximum displacement of the vessel wall and the inner lining of the thrombus for a configuration with a symmetrically located lumen of the vessel (top left), with a shift of the lumen from the spine (top right), with a shift of the lumen closer to the spine (bottom left) and for shifting the lumen longitudinally relative to the vessel (bottom right). Also in the figures there are corresponding schemes for the location of the lumen.

**Fig 15:**
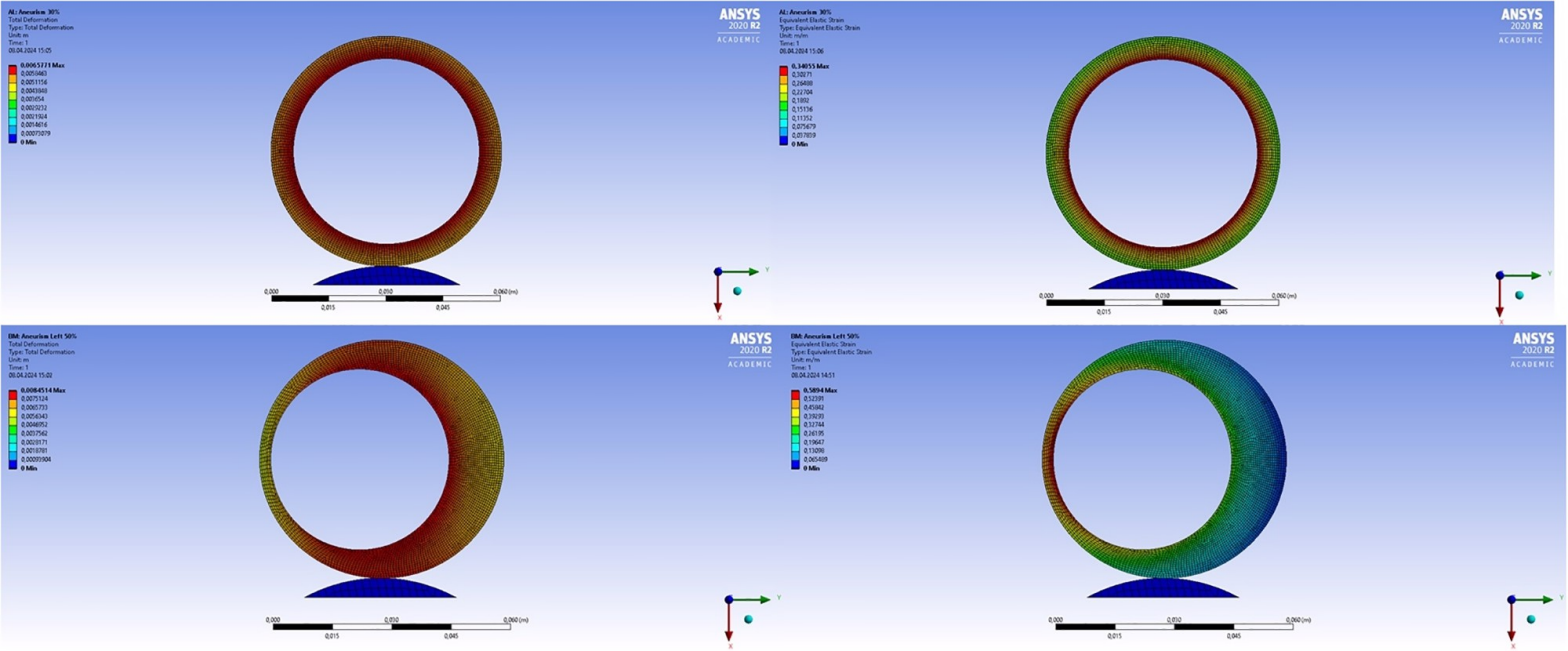
Example of changing the position of the wall. Distribution of calculated stresses (left) and maximum displacements (right) for a configuration with a symmetrically located vessel lumen (top) and for displacement of the lumen longitudinally relative to the vessel (bottom).

As we can see, in general, the results of numerical modeling correlate with the results of ultrasound monitoring: the maximum deformations for any of the considered thrombus configurations for the internal lining of the thrombus exceed the deformations of the aneurysm wall in absolute value. When the lumen of the aneurysm is filled with a thrombus between 60% and 80% the values of the maximum deformation of the thrombus wall and the aneurysm are interchanged (Fig 14). Similar situation is also observed for cases of asymmetrically located thrombi, displaced to the left or to the front of the spine. In such situations, with an increase in the volume of thrombus masses, the direction of increase/decrease in the maximum deformation for the thrombus wall and the aneurysm are opposite. An example of the distribution of strains and stresses for the symmetrical and asymmetric case of the location of an intraluminal thrombus is shown in (Fig 15).

**Fig 16:**
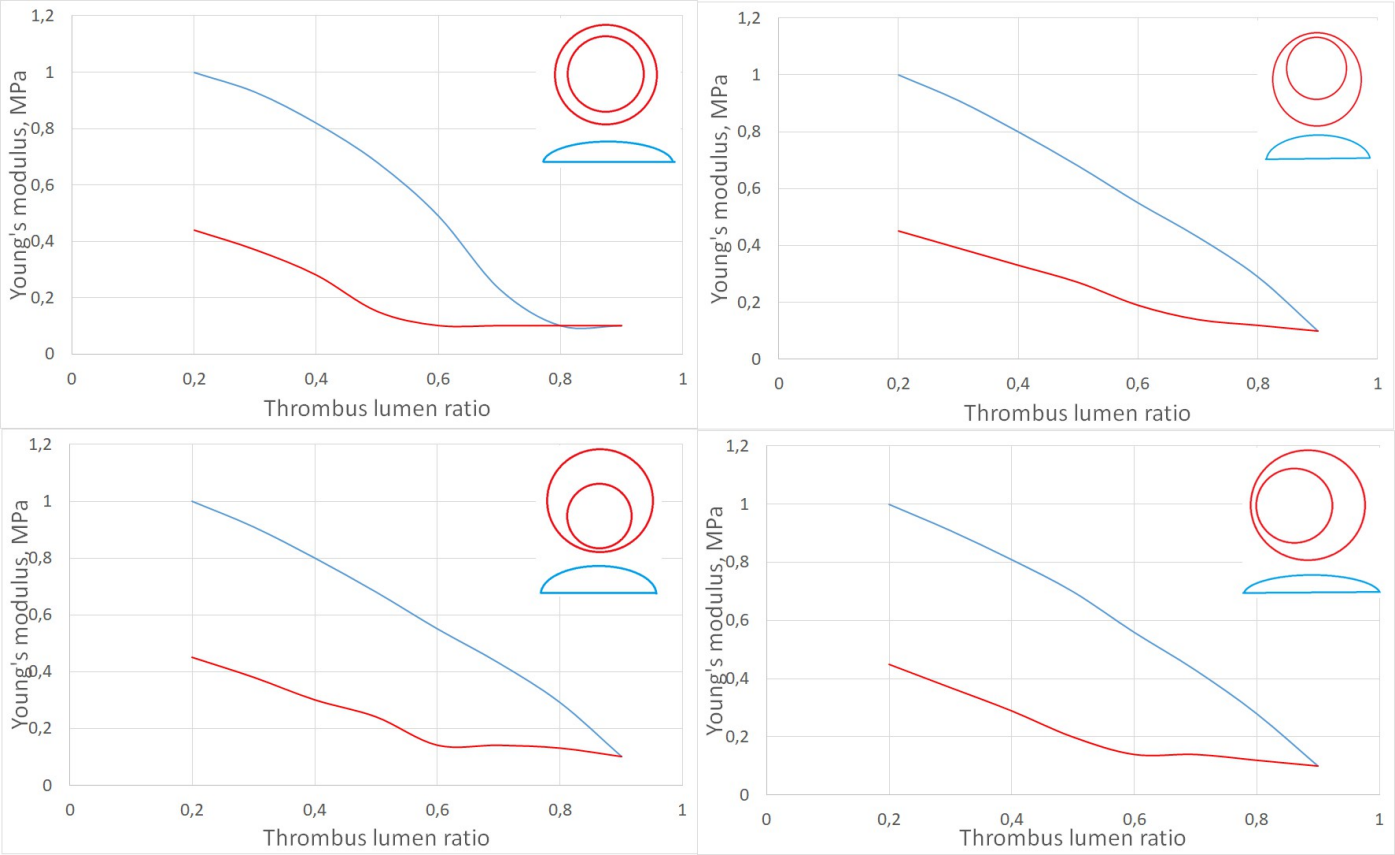
Digital aortic wall Young’s modulus. Dependence of Young’s modulus on the amount of thrombus in the vessel (blue line—minimal deformation threshold, red line—critical deformation threshold) for a configuration with a symmetrically located lumen of the vessel (top left), with a shift of the lumen from the spine (top right), with a shift of the lumen closer to the spine (bottom left) and for shifting the lumen longitudinally relative to the vessel (bottom right). Also in the figures there are corresponding schemes for the location of the lumen.

Next, we performed a series of numerical calculations, the purpose of which was to determine the minimum threshold of the Young’s modulus of the aneurysm wall, which allows a solution to exist while maintaining the density of thrombus masses and pressure from the lumen of the vessel. As a result of numerical simulation, two distributions of the values of the Young’s modulus of the wall were obtained for all configurations. Examples of the obtained calculated stresses and displacements of the thrombus and the wall are shown in Fig 15. After processing all cases of the location of the lumen in thrombus masses and the amount of thrombus (thrombus lumen ratio), it was found that the most rupture-resistant configuration is the case with the central location of the lumen, since the critical deformation threshold (values of the Young’s modulus of the aneurysm wall, a decrease in which by 0.01 MPa leads to all cases to the collapse of the numerical calculation) reaches the value of the Young’s modulus of the aneurysm wall of 0.1 MPa with the least amount of thrombus in the vessel. It makes no sense to reduce the Young’s modulus of the wall to less than 0.1 MPa, since such parameters go beyond the known limits of the elasticity of the vascular wall. Displacement of a thrombus in any direction leads to an increase in the risk of rupture of the aneurysm wall, since the critical deformation threshold continues up to the thrombus lumen ratio of 80%. The results presented at the Fig 16.

**Fig 17:**
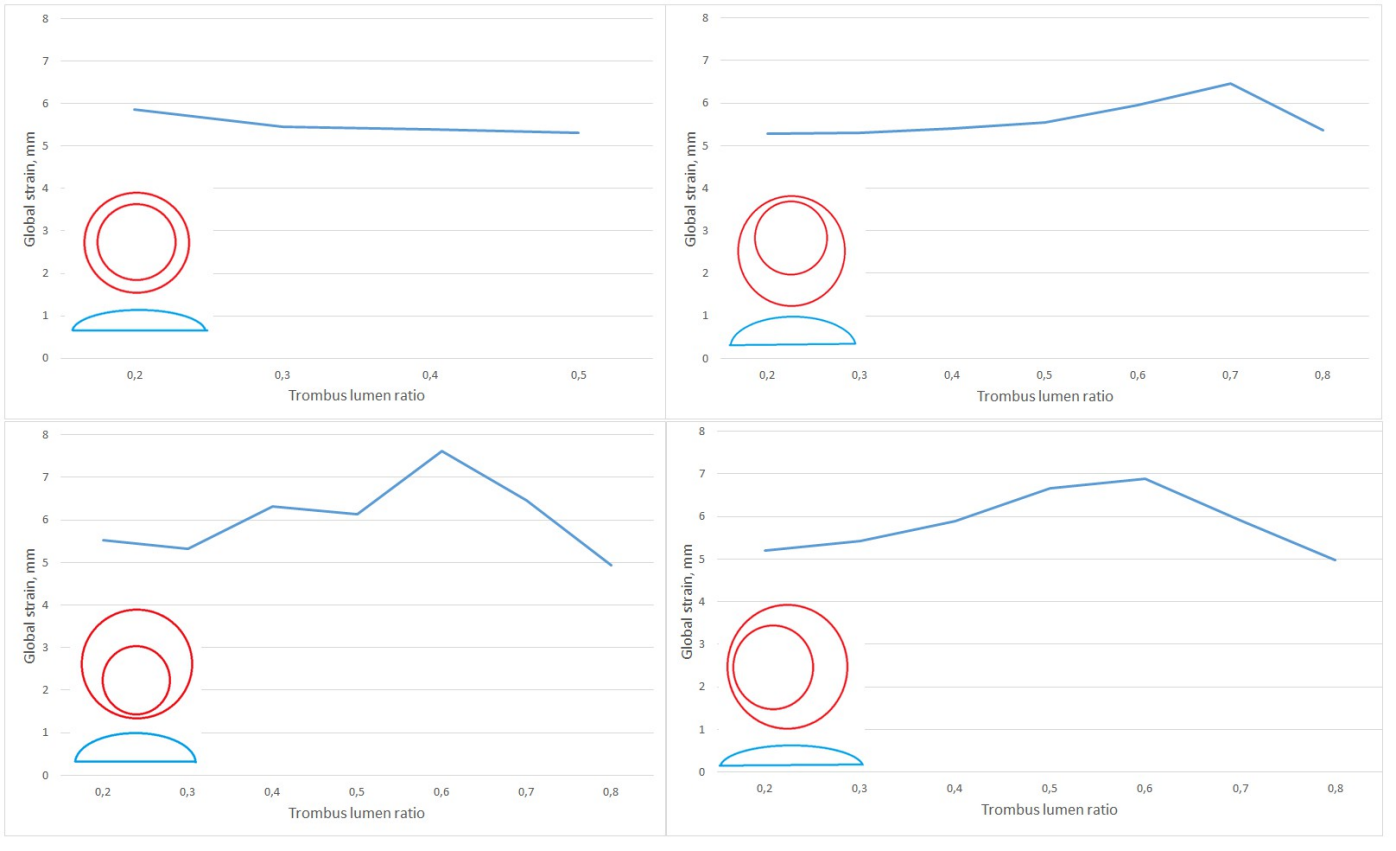
Average digital aortic wall strain. Results of numerical calculations (averaged values of aortic wall displacements) for different types of intraluminal thrombus location.

Also, for all configurations, the average displacement of the wall of the aortic aneurysm relative to the starting position was calculated. Graphs are shown in Fig 17. According to these data, it can be seen that in the case of a central location of the lumen, the average displacements of the aneurysm wall decrease with an increase in the number of thrombus inside the aneurysm, but by no more than 10%. In the case when the lumen is displaced from the spine, the average displacement of the aneurysm wall increases with an increase in the thrombus lumen ratio, but after 70% filling of the aorta, the displacements decrease, which is a stabilizing factor and has medical confirmation. For other cases, such a change in values occurs after the thrombus lumen ratio value of 0.6. In addition, it is worth paying attention to the nature of the intersection of the curves of the minimum Young’s modulus and the prediscontinuity one. If for the case of a symmetrical thrombus it has the character of a tangent, i.e. approach to the state of prerupture is continuous and monotonous, then the nature of the intersection of these curves for the variant of a thrombus with a displaced eccentricity is completely different. There is a sharp intersection in an area that is practically not relevant from the point of view of observations (the lumen is almost completely closed), which contains patients with almost complete obstruction of the aorta and already requiring surgery to restore blood flow to the lower extremities.

## Discussion and perspectives

This study occupies an intermediate role between predictors of AAA growth and rupture. While it has been shown that various factors [27] may be predictors of growth and are not predictors of AAA rupture, there are works in which a place has already been given to intraluminal thrombi and the influence of the volume and localization of thrombus masses on the status of AAA rupture has been assessed [18]. However, not all variations of thrombus locations have been thoroughly investigated in prior studies. Our study addresses this gap by presenting relevant statistics. Previously, studies that evaluate changes in the lumen of the aneurysm during pulsations, used MRI [28]. However, this protocol is highly demanding in terms of the use of MR resources and is unlikely to become a routine practice. In addition, this approach does not consider the role of thrombotic masses, which can occupy a large part of the AAA space and, therefore, significantly change its biomechanics. The accuracy and reproducibility of ultrasonic monitoring data is one of the main disadvantages of this method, which is widely discussed [29].

During the study, we demonstrated the accuracy of the data concerning the maximum diameter and percentage of thrombus masses within the AAA. Despite their initial acquisition through ultrasound diagnostics, these values were confirmed to correlate with the analysis of CT images from the studied patients. The authors of this work considered a small number of patient-specific configurations and the only mechanism of contact of thrombus zones with different densities, which in no way allows drawing general conclusions. That is why we chose the tactics of performing idealized calculations, since in such configurations the geometric features inherent in a particular patient are completely excluded. Our specific focus was on the intraluminal thrombus (ILT) parameters, particularly the thrombus location. In addition to [18] we also considered the displacement of the thrombus relative to the axis of symmetry of the body passing through the spine, which was not considered earlier in the literature. Nonetheless, we found support for our hypothesis in the paper [30] indicating heightened inflammation on the aneurysm wall under the parietal thrombus due to hypoxia [31] with secondary degradation of the extracellular matrix. Inflammation likely plays a role in the thrombus structure’s deterioration, subsequently altering the hydrodynamic load on the underlying vascular wall and increasing the risk of rupture. However, this work does not take into account the quantitative indicators of thrombus masses, and only concludes that their presence has a significant effect on the rate of AAA growth. Thrombus asymmetry can cause desynchronized deformations of the inner wall. In cases where the density of the thrombus and the aneurysm wall are comparable, the oscillations of the system under the action of a pulse wave will likely be unidirectional. At the same time, with a strong difference in the density of the thrombus [19, 20] and the aneurysm wall, the movement of the inner wall of the thrombus will be directed towards the center of the lumen due to hydrodynamic suction, otherwise a part of the thrombus must inevitably break away from the main mass due to the continuity of the blood flow in the aneurysm lumen. In cases where the movements of such a dense thrombus become unidirectional, along with the aneurysm wall (radially outward), we presume that the thrombus integrity is already compromised. Otherwise, conflicts with the flow continuity within the AAA arise once more.

The advantage of this work in comparison with those already published is the presented new method for non-invasive ultrasound diagnostics of aortic wall deformations in healthy aorta and abdominal aneurysm. In combination with a criterion based on the results of numerical modeling of an idealized aortic aneurysm with an intraluminal thrombus, it allows not only qualitative but and quantitative risk analysis of aneurysm rupture and thromboembolic complications. A good correlation was shown between the results of measuring the linear dimensions of the aneurysm and the operator-independent CT technique.

The proposed numerical model’s advantage lies in its simplicity, even though it encompasses more potential cases of thrombus location than previous publications. In addition, the simplicity of the model made it possible to provide a qualitative substantiation of the causes of thromboembolism or rupture for asymmetric and symmetric cases of thrombus growth, and the quantitative boundary of such phenomena coincides with the results of ultrasound measurements. The study, however, has a number of limitations. The patient sample size is insufficient to establish the statistical significance of the results. Additionally, the study excluded patients with aortic tear and rupture. Furthermore, only the transverse strain of the aorta was assessed in the area of its maximum expansion. Consequently, desynchronized deformation curves were obtained only from a limited area of the aorta, not allowing the assessment the entire aneurysmal sac.

In the numerical modeling aspect, averaged rather than personalized data on the viscoelastic characteristics of the thrombus, aorta and hemodynamic parameters were used, as well as averaged data on the strength properties of the vascular walls of the studied patients [24]. Similar approaches are currently actively used in averaged modeling of aortic hemodynamics with heterogeneous walls [32]. In reference to related research, a parallel stage of the study is discussed in [18], which examines a database of patients with aortic aneurysms and highlights the primary distinctions in the strength characteristics of aneurysm-affected aortas and those with other vascular tissue lesions in the abdominal segment. In [28], the authors came closest to the problem we are studying, although their focus centered on a small, patient-specific sample size and concentrated on areas of calcification and their impact on the mechanical behavior of aortic aneurysm tissue. In contrast, our proposed approach places more emphasis on the interaction between aortic tissue and intraluminal thrombus to identify the risks of thromboembolism and rupture. Nevertheless, both approaches and their combination deserve further attention, since both the already published works and this study contain significant limitations that do not allow us to fully reliably and in detail describe the dependence of the mechanical behavior of the aortic aneurysm depending on its structural composition.

## Conclusion

We have shown for the first time that asymmetric aortic dilation with asymmetric remodeling of the lumen by thrombotic masses are the most likely predictors of a decrease in the strength of the vascular wall. It’s possible that differences in elastic deformation between the vascular wall free from thrombotic masses and the area covered by the thrombus may prompt the mobilization and detachment of the thrombus from the vascular wall, potentially leading to thromboembolic complications. This results in heightened pressure in the AAA region and ultimately a rupture of the aortic wall. Considering the limitations acknowledged in our study, the model developed here offers valuable insights into predicting thromboembolic complications and identifying aneurysm cases that pose a genuine high-risk potential for rupture by relying on general principles of mechanics, supported by reliable experimental data. Additionally, for the first time, using numerical finite element modeling, a substantiation of the effect of an asymmetric thrombus as a predictor of hemodynamic complications was given, while the basis for the numerical problem conditions is the measurement data of the aortic deformation during one cardiac circle of 29 patients with an aneurysm and 18 healthy volunteers. Thus, our work marks the initial steps toward implementing the speckle tracking technique in ultrasound diagnostics of the abdominal segment of the aorta. As this method gets further refined and becomes more widely applied, it has the potential to unveil numerous new physiological effects. At the same time, the advancement of methods for patient-oriented modeling of the contact interaction of the AAA wall, intraluminal thrombus and blood flow will significantly reduce the number of clinical cases required to collect evidence-based statistics.
